# RETRACTED: Hong et al. Wogonin Suppresses the Activity of Matrix Metalloproteinase-9 and Inhibits Migration and Invasion in Human Hepatocellular Carcinoma. *Molecules* 2018, *23*, 384

**DOI:** 10.3390/molecules31040608

**Published:** 2026-02-10

**Authors:** Ming Hong, Honghui Cheng, Lei Song, Wencai Wang, Qi Wang, Donggang Xu, Weiwei Xing

**Affiliations:** 1 Institute of Clinical Pharmacology, Guangzhou University of Chinese Medicine, 12 Jichang Road, Guangzhou 510405, China; songyt1994@163.com (L.S.); wencai.wang@qmul.ac.uk (W.W.); wangqi@gzucm.edu.cn (Q.W.); 2 College of Mechanical Engineering, Yangzhou University, 88 South University Ave., Yangzhou 225009, China; hhcheng@yzu.edu.cn; 3 Department of Genome Engineering, Beijing Institute of Basic Medical Sciences, Taiping Road 27, Beijing 100850, China; xudg@bmi.ac.cn

The journal retracts the publication “Wogonin Suppresses the Activity of Matrix Metalloproteinase-9 and Inhibits Migration and Invasion in Human Hepatocellular Carcinoma” [1], cited above.

Following publication, concerns were brought to the attention of the Editorial Office regarding the integrity of an image presented in this publication [1].

In accordance with the journal’s standard editorial procedures, the Editorial Office and the Editorial Board conducted an investigation, which identified indications of inappropriate editing and partial duplication of panels within Figure 1, as well as between panels in Figure 1 and panels presented in a previous publication [2], produced by a different research group and presenting different experimental conditions. The authors did not provide a comment on this concern. Consequently, the Editorial Board has lost confidence in the reliability of the findings and has decided to retract this publication, as per MDPI’s retraction policy.

This retraction was approved by the Editor-in-Chief of the journal *Molecules*.

The authors have been informed of this retraction but did not provide a comment on this decision.
